# Operative Treatment of Tuberculous Joint Diseases

**Published:** 1894-01-13

**Authors:** 


### CURRENT MEDICAL TOPICS.

### OPERATIVE TREATMENT OF TUBERCULOUS JOINT DISEASES.

MANY names have been given to this malady, many are the treatments to which it has been subjected. but it is only since it has been recognised as a form of tuberculosis, and since tuberculosis has been recognised as an infective disease, that any true conception has been possible as to why in some cases cure results, and in others treatment has proved so absolutely unsuccessful. The aim of the surgeon is twofold; firstly, to get rid of the diseased parts; secondly, to do so without the distortion which almost always accompanies natural cure.

From the earliest times it has been observed that a large proportion of the cases tend to get well if they can but be kept quiet. Hence the great tribe of splints which have been in use from time immemorial; hence also the dislike among many of having recourse to operative proceedings.

Tuberculosis we know to be a disease of enfeebled tissues, a disease each focus of which, charged as it is with bacilli, tends to become surrounded by a zone of inflammatory products, into which it grows, and by means of which it spreads. Although, then, we can do but little to the bacilli, we can do much to check the spread of inflammation caused by the movement of these diseased tissues, and so it is that, under the influence of the rest, secured by properly contrived splints, the tubercular process, while running its course in the parts already affected, ceases to extend to others, and thus a cure results. For in truth, the course of the tubercular process in any single point is but a short one, the long drawn out history of cases of consumption or of joint disease depending on the gradual involvement of fresh parts.

Treatment by splints, then, is not a mere dark and mysterious proceeding handed down to us from our grandmothers, but is, or ought to be, a treatment applied with most painstaking care for the definite purpose of ensuring absolute immobility. Splints and plasters are unmitigated evils, only adding to the weight of the limb and the discomfort of the patient, unless they fulfil their one single purpose, which is the preduction of immobility; if they secure this, however, they often lead to cure. Hanging as a dark shadow, however, over this treatment by rest and waiting, is the constant fear lest the whole process should be rendered useless by the development of tuberculosis in other organs. It has always been recognised that strumous joints, and especially such as involved confinement to the house, often lead to consumption, and now that we know that the joint diseases themselves are tubercular, the reason of the association is plain enough. It is often suggested as a reason for not operating that even after operation such a long time is required for complete recovery that perhaps, even without operation, so long a rest might lead to cure. Waiting for cure, with the tuberculosis in one, is, however, a far more dangerous process than waiting for strength after it is removed.

Suppose then it is decided that an operation is to be performed, what operation is to be done?

If details are to be taken into account the variety of operations is almost infinite; they may, however, be grouped under three headings, amputation, excision, and arthrectomy.

Now in the good old times amputation was the almost universal operation, and by it a great number of useful lives were saved; unfortunately by it also a great number of useful limbs were lost. Still it had great advantages. After years of misery, patients were rapidly restored to useful health; it frequently snatched people apparently from the very jaws of death, and it removed the whole of the disease definitely and completely. Even now amputations often have to be performed, but chiefly as a last resort, when it is clear that smaller measures would not succeed, or when the part to be saved is hardly worth the time required to save it, as in some portions of the foot.

The substitution of excision for amputation was, in some hands, and in regard to some joints a great advance in surgical procedure. In the elbow the results were good in a large proportion of the cases; in the knee good in a certain proportion, but disastrous in many; in the ankle bad; in the hip variable in different hands; and it was difficult to say why an operation, the anatomical details of which could be so accurately laid down, should be so uncertain in results. It was not, in fact, at first recognised how widespread the disease was apt to be, and how essential was the removal of the whole of it. The ends of the bones were removed with great nicety and skill, but quantities of diseased tissue were sometimes left, and thus failure resulted.

With the discovery of the *bacillus tuberculosis*, however, there came new light. As soon as it was seen that all the pulpy gelatinous material, into which the tissues of the joint had degenerated, was cram full of bacilli, it became obvious enough that every bit of it which was left might become the germ of a recurrence, and that the essential thing in the operation was, not the accurate removal of slices of bone, but the careful extirpation of the whole of the disease in soft tissues as well as in hard. In the modern excision then the removal of the bone is but a detail in the sometimes much larger proceedings necessary for the removal of the whole mischief, and the operation has thus become an arthrectomy, plus, a removal of certain pieces of bone.

What do we mean by an arthrectomy? Well, we must not be tied down by etymology. It does not mean merely cutting out a joint, but rather the cutting out of all the diseased tissue of which the joint is the centre, be it bone, or cartilage, or pulpy degeneration of ligament or synovial membrane, and as far as possible leaving all the rest. No doubt to the eyes of many the scraping or erasion of diseased joint surfaces, instead of the resection of the bone, appeared at first a further step in the simplification of surgery. Why indeed remove a slice of bone when only a small patch of it is diseased? Hence the days of scrapings and small incisions, and hence probably the failures. Many surgeons even now will say, speaking of a case, that nothing short of an excision is likely to be of any use, as if excision were the major operation; so far are they from the standpoint of others who look on it as but an imperfect proceeding, an incomplete removal of the disease. There can be no doubt that the success of arthrectomy, in the hands of its modern advocates, depends on it being made a very major operation indeed, in which by knife and scissors, by gouge and spoon, and even by saw, all unhealthy tissue is got rid of, and in which the incisions are large and free, so as to expose the whole joint surface, even at the expense of dividing, may be, the patella, the olecranon, or the tendons in front of the ankle, or of slicing off the top of the astragalus.

From the point of view of those who advocate arthrectomy there are but few cases in which a classical excision is justifiable. It either does too little or too much. At the same time, it is clear that a modern excision, in which, as well as bone, all diseased structures are removed, is really an arthrectomy, with the addition of certain definite incisions of bone with certain definite ulterior aims—in the knee, for example, for the sake of ensuring bony union; in the elbow for the sake of preventing it.

Looking at matters in this light, it is probable that the choice of operation in the future will lie between amputation and arthrectomy, and that the success of the latter will depend on its thoroughness; but it may well be opined that the growing success of this operation will introduce a new factor into the consideration of such cases. Precisely the same feeling which held surgeons back from amputation if it could possibly be avoided, has deterred them from doing early excisions because, whether the disease were much or little, the full operation must be done. But in arthrectomy things are different; the incisions may not vary, but the amount of tissue removed, and the resulting disability, will depend greatly on the extent of the disease, and thus the tendency will doubtless be to do the operation much earlier than has hitherto been the practice, and so to avoid many risks.

